# Assessment of home storage of antimicrobials and its predictors in Mecha demographic surveillance and field research center: a cross-sectional study

**DOI:** 10.1186/s12879-023-08227-7

**Published:** 2023-05-03

**Authors:** Endalew Gebeyehu, Misgan Ararsie

**Affiliations:** Department of Pharmacy, College of Medicine and Health Sciences, Bahir Dar University, P.O.Box 79, Bahir Dar, Ethiopia

**Keywords:** Home stored antimicrobials, Predictors, Households, Mecha

## Abstract

**Background:**

Home storage of antimicrobials is a worldwide practice. Irrational storage and inappropriate use of antimicrobials should get special attention in low-income countries due to limited information, knowledge, and perceptions. This study was conducted to survey home storage of antimicrobials and assess its predictors in Mecha Demographic Surveillance and Field Research Center (MDSFRC), Amhara region, Ethiopia.

**Methods:**

A cross-sectional survey was conducted on 868 households. Predeveloped structured questionnaire was used to collect data on sociodemographics, knowledge on antimicrobials and perception about home stored antimicrobials. Data was analyzed using SPSS version 20.0 to execute descriptive statistics, and run binary and multivariable binary logistic regression. P-value < 0.05 was considered significant at 95% confidence level.

**Results:**

The total number of households included in this study were 865. Female respondents represent 62.6%. The mean age (±) of respondents was 36.2 (± 13.93) years. The mean family size (±) of the household was 5.1 (± 2.5). Nearly one-fifth (21.2%) of the households stored antimicrobials at home with a condition similar to any household material. Most commonly stored antimicrobials were: Amoxicillin (30.3%), Cotrimoxazole (13.5%), Metronidazole (12.0%), and Ampicillin (9.6%). The most common immediate source of home stored antimicrobials was discontinuation of therapy (70.7%) either from symptomatic improvement (48.1%) or missing doses (22.6%). Predictors of home storage of antimicrobials with corresponding p-value were: age (0.002), family size (0.001), education status (< 0.001), home distance from the nearby healthcare institution (0.004), counseling while obtaining antimicrobials (< 0.001), knowledge level on antimicrobials (< 0.001), and perception of home stored antimicrobials as a wisdom (0.001).

**Conclusion:**

Substantial proportion of households stored antimicrobials in a condition that may exert selection pressure. To reduce home storage of antimicrobials and its consequences, stakeholders should give due attention to predictors variables related to sociodemographics, level of knowledge on antimicrobials, perception of home storage as a wisdom, and counseling service.

## Introduction

Antimicrobials have played a significant role in improving public health by reducing the number of deaths from infectious diseases which were previously incurable or fatal [1, 2]. Excessive and uncontrolled use of antimicrobials has been reported to accelerate antimicrobial resistance [1, 3]. On top of that, in most countries, the general public have many preconceived ideas concerning antimicrobials and their effects [4]. Many people still believe that antibiotics kill viruses and effective against colds and flu [5]. Currently, an increasing number of patients are infected with resistant microorganisms resulting in longer, more complicated courses of treatment, a greater risk of death and extra costs for healthcare system [6, 7].

Self-medication practice is widely accepted and successfully integrated into many healthcare systems throughout the world [8]. In most economically deprived countries many prescription drugs are dispensed without prescription due to lack of awareness, poor practice of the guidelines, incorrect perceptions, and lack of accountability of both users and suppliers [4]. In Ethiopia, self-medication practice with antimicrobials ranges from 38.9 to 67.3% mainly for its low cost [9–11]. Parallel to this, dispensing antimicrobials without prescription is also reported to vary from 67.7 to 94.4% [12–14]. These reports indicate existence of inappropriate use of antimicrobials in the country. Even though, self-medication, dispensing without prescription and home storage of antimicrobials are common practices, they are not recommended by Ethiopian guidelines [15, 16]. To minimize risks associated with home storage of antimicrobials, the country should put community strategies against antimicrobial resistance through encouraging the prudent use of these substances [17]. This study was conducted to survey home storage of antimicrobials and assess its predictors in the community since there was no documented data in the field research center where the study was initiated.

## Methods

### Study design and period

A cross-sectional study was carried out to assess storage of antimicrobial at home and to identify predictors of the storage practice with data gathered in the community from March to May 2020.

### Study area and population

The study was conducted in Mecha district (Wereda) in West Gojjam zone of Amhara Regional State. Merawi is the capital of Mecha district and 34 Km far from Bahir Dar, the capital city of Amhara Regional State. The district has 4 urban and 39 rural kebeles. Kebele is the lowest administrative unit in the country. Bahir Dar University has established Mecha Demographic Surveillance and Field Research Center (MDSFRC) in Mecha district. MDSFRC has a total of 10 kebeles that contains 19,200 households and a population of 81,000 people. Heads of households or family members fulfilling inclusion criteria of respondents in selected kebeles of MDSFRC were the study population. Those who are able to communicate with the local language and age above 18 years were included.

**Fig. 1:**
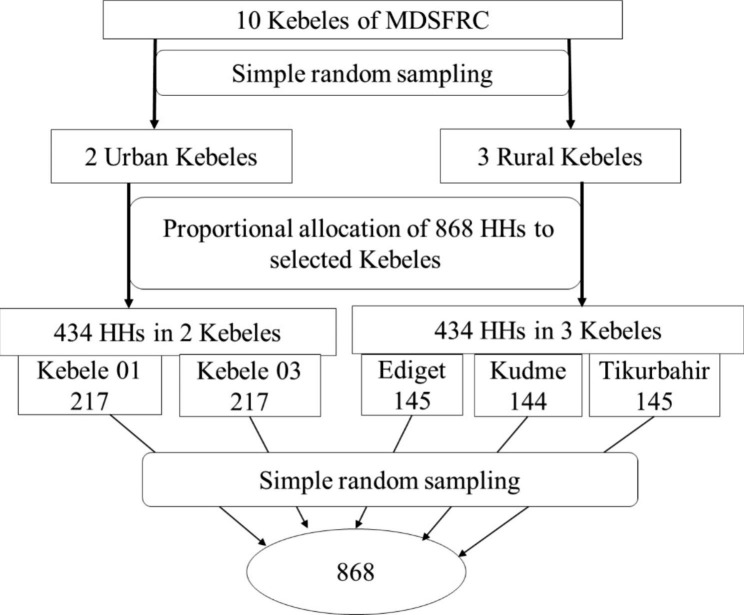
Schematic representation of proportional allocation calculated sample size of households (HH) to randomly selected Urban and Rural Kebeles

**Sample size determination and sampling technique**: The minimum sample size for this study was calculated using one population proportion formula by considering the following assumptions: P (50%) [assumed practice of keeping antimicrobials at home since there was no national or local previous data], 95% - Z-score (1.96), d (0.05), 2 design effect, 13% non-response rate. Based on these assumptions the total sample size computed was 868. Of the total 10 kebeles under field research center; two urban kebeles (Kebele 01 and Kebele 03), and three rural kebeles (Ediget, Kudmi and Tikurbahir) were selected by taking into account of the total sample size and number of households present in rural and urban areas in the respective Kebeles, as shown in Fig. 1. Equal number of households were selected in urban and rural kebeles to minimize effect of residence on dependent variable. The household records of the field research center was used as a sampling frame to undertake systematic random sampling.

### Data collection tool and Procedure

The data collection tool was developed by considering reviewed literatures, features of the respondents, and the local context. Besides structured questions, the data collection tool was made to consist of information about stored medications including antimicrobials. Pretest was done to assess predeveloped structured questionnaire in an unselected kebele. The structured questionnaire comprises of sociodemographics of study respondents, knowledge on antimicrobials, and perception about home stored antimicrobials. When data collection was impossible at a selected household, a house next to the selected house was involved in the survey. During data collection, respondents were requested to show all available drugs to complete the checklist of stored drug information which includes categorization of drugs into antimicrobials and non-antimicrobial drugs, name of drug, the quantity, drug formulation, expiration date, and placement of the stored drugs. After drug related information was noted, data collectors gathered more data on; whether the drugs were currently in use or not, where they obtained the drug(s), and what to do with them were collected. Although the standard measurement of distance is kilometer, as estimate of home distance from the nearby healthcare institution was measured in walking hour expecting most respondents could be unaware of kilometer.

### Operational definitions

i. **Antimicrobials**: In this manuscript the word antimicrobials refers to all pharmaceutical preparations used to treat infections other than helminthiasis.
ii. **Home Storage of Antimicrobials**: It is the retention of one or more antimicrobial(s) at home intentionally and/or unintentionally which could be obtained from discontinuation of drug therapy due to symptomatic improvement, forgetfulness, dosage changes, side effect intolerance, expiration, death of patients and/or deliberately purchased. Antimicrobials on use during data collection period were not considered as kept if obtained through professional prescription, however antimicrobial obtained by self-initiated interest was considered as kept.
iii. **Over-the-counter (OTC) medicines**: are medicines that may be sold (dispensed) directly to a consumer without possessing a valid prescription.
iv. **Level of knowledge on antimicrobials**: it is the knowledge level of respondents categorized based on Likert scale out of the responses from 10 questions.
v. **Perception about home stored antimicrobials**: the feeling and the value that respondents give to home stored antimicrobials.
vi. **Counselling while receiving antimicrobials**: receiving any type of information about antimicrobial while getting the drug into the hands of the respondents.

#### Data quality management

Pretest of the questionnaire was done in study area kebeles which were not selected for data collection. Training was given to data collectors, and daily supervision was done by principal investigator to check completeness of the questionnaire.

#### Data analysis procedures

Although a total of 868 households were surveyed, the data entered for analysis of this study comprises of 865 household due incompleteness of the data of three households. Data was entered and analyzed using SPSS statistical package version 20.0. When the frequencies of categories of original data collection tool were found insufficient for data analysis re-categorization was conducted with logical reason. Descriptive statistics (percentages, means and standard deviations) of the collected data was calculated to describe data. Knowledge level of respondents on antimicrobial was summarized by computing their correct answers of 10 questions and categorized by using Likert scale knowledge into very high, high, average, low and very low. Binary and multivariable logistic regression models were used to identify factors associated with practice of keeping antimicrobials at home. All variables with p-value <0.25 on bivariate logistic regression were analyzed on multivariable logistic regression based on recommendation given elsewhere [18]. Before running multivariable logistic regression, variance inflation factor (VIF) was computed to test multicollinearity of independent variables. The maximum VIF among the variables was 3.5 which is lower than 5, the maximum tolerable VIF for analysis. Crude odds ratio with 95% confidence interval was calculated to measure the association of dependent and independent variables. P-value < 0.05 was considered significant.

#### Ethical considerations

The study was conducted according to the Declaration of Helsinki after getting permission from the Ethical Clearance Committee of College of Medicine and Health Sciences, Bahir Dar University with a Ref. No: 1/503/109. On presenting the ethical clearance and our request, Amhara Regional State Health Bureau provided official letter to Mecha District Health Office to conduct the study in the selected Kebeles. Written informed consent was obtained from each respondent after briefing the objectives. Confidentiality and privacy were assured for information collected from households.

## Results

### Features of stored antimicrobials at home

Of the total 865 surveyed households included in this study, stored antimicrobials were found in 183 household and all medications including antimicrobials were identified in 275 households. The percentage of households that store antimicrobials was 21.2% (183/865) which accounts 66.5% (183/275) of all the medications stored in households as shown in Table 1. Due to storage of more than one type of antimicrobial at a home, there were 208 items of antimicrobials from 183 households. Amoxicillin was the most commonly stored antimicrobial (30.3%) followed by cotrimoxazole (13.5%), and metronidazole (12.0%). The primary source of home stored antimicrobials was prescription (69.2%), whereas 29.3% of antimicrobials were purchased directly from pharmacy. Discontinuation of therapy from symptomatic improvement (48.1%) and missing doses (22.6%) was the main trigger for home storage of antimicrobials followed by deliberate reservation (23.1%). Nearly 90% of stored antimicrobials were capsules (48.6%) and tablets (38.9%). Common household material storage areas were utilized for home storage of 90% (90.2%) of antimicrobials, where 64.5% were on shelves and tables and 25.7% in boxes and hand bags.

**Table 1:** Some characteristics of stored antimicrobials at home in Mecha demographic surveillance and field research center, Ethiopia, March to May 2020 (N = 183)^a^

Characteristics	Frequency (Percentage)
Stored antimicrobials at home	183(21.2)
All medications stored at home	275(31.8)
Type of stored antimicrobials at home:	
Amoxicillin	63(30.3)
Cotrimoxazole	28(13.5)
Metronidazole	25(12.0)
Ampicillin	20(9.6)
Ciprofloxacin	14(6.7)
Cloxacillin	13(6.3)
Chloramphenicol	13(6.3)
Doxycycline	13(6.3)
Tetracycline	6(2.9)
Tinidazole	4(1.9)
Erythromycin	4(1.9)
Others^b^	4(2.4)
Triggers of storage of antimicrobials at home:	
Left from discontinuing on symptom improvement	100 (48.1)
Deliberately reserved	48 (23.1)
Left from missed doses	47 (22.6)
Left from discontinuing on health professional decision	7 (3.3)
Others including side effects	6 (2.9)
The way the antimicrobials were obtained:	
Prescribed by prescribers	144 (69.2)
Purchased directly from pharmacy	61 (29.3)
Others (borrow)	3 (1.5)
Type of formulation of stored antimicrobials:	
Capsule	101 (48.6)
Tablet	81 (38.9)
Bottle	23 (11.1)
Others (Drop/Ointment/Paste)	3 (1.4)
Expiration date of stored antimicrobials at home at the time of data collection:	
Not expired	135 (64.9)
Expired	60 (28.8)
Not visible	13 (6.3)
Site of placement of stored antimicrobials at home:	
Elsewhere on shelf/table	118 (64.5)
In Box/Bag	47 (25.7)
Others (Plastic bag, purse, cartoon)	18 (9.8)
What to do with stored antimicrobial (Desire):	
Dispose	77 (42.1)
Preserve for future use	69 (37.7)
Don’t know what to do	37 (20.2)

^a^Represents the number of households that store at least one antimicrobial; ^b^Others include Azithromycin, Gentamycin, Norfloxacin, and Rifampicin

### Sociodemographic, Knowledge and Perception of Respondents

Data collected from a total of 865 households was included in this study that make the response rate households to be 99.6% (865/868). The summary of sociodemographic, knowledge, and perception of respondents representing the households was shown in Table 2. The sex nearly two-third (62.5%) of the participants was females. The mean age of the study participants was 36.2 years (SD ± 13.93). The mean family size of the households was 5.1 (± 2.5 SD). Almost two-third (63.5%) of the study participants were married. Education level of 44.4% of the participants was unable to read and write. Occupation wise nearly half (48.8%) were farmers. Only 13.7% of participants were employees with regular monthly salary, whereas the remaining is composed of housewife, merchants, students, and daily laborers. Nearly one-third (32%) participants reported presence of a university graduate in the family. Nearly three-quarters (74.2%) of the participants were within a distance of less than 1-hour from the nearest health center in walking hours. Attainment of counseling while obtaining antimicrobials was 44.4% indicating that more than half obtained antimicrobials without gaining counselling service. Summary of knowledge of respondents based on 10 questions showed that about half was below average score (50%). More than half (51.5%) of the respondents either strongly agree or agree that stored antimicrobials at home do not need any special attention and considered as medications. Nearly 40% of the respondents either strongly agree or agree with a statement that describes storage of antimicrobials at home as a reserve is a sign of wisdom. Nearly 25% of respondents either strongly agree or agree with a statement that says, it is better to use any available antimicrobials at home to a sick in the family before vising healthcare institutions. Approximately 30% of the respondents either strongly agree or agree with a statement that says, antimicrobials stored elsewhere at home has nothing to do with antimicrobial resistance.

**Table 2:** Summary of sociodemographic, knowledge, and perception of respondents on storage of antimicrobial at home in Mecha demographic surveillance and field research center, Ethiopia, March to May 2020

Variables and categories	Respondents representing HHs (N = 865)			
	Frequency^a^	Stored^a^ (n = 183)	Not Stored^a^ (n = 682)	Storage in %
Place of residence:				
Rural	435 (50.3)	84 (45.9)	351 (51.5)	19.3
Urban	430 (49.7)	99 (54.1)	331 (48.5)	23.0
Sex:				
Male	323 (37.4)	71 (38.8)	252 (37.0)	22.0
Female	542 (62.6)	112 (61.2)	430 (63.0)	20.7
Age (mean ± SD; 36.2 ± 13.93)				
< 25	211 (24.4)	29 (15.8)	182 (26.7)	13.7
25–34	200 (23.1)	43 (23.5)	157 (23.0)	21.5
35–44	232 (26.8)	60 (32.8)	172 (25.2)	26.9
45 − 54	117 (13.5)	28 (15.3)	89 (13.0)	23.9
> 54	105 (12.1)	23 (12.6)	82 (12.0)	21.9
Family size:				
1–2	135 (15.6)	14 (7.7)	121 (17.7)	10.4
3–4	249 (28.8)	41 (22.4)	208 (30.5)	16.5
5–6	230 (26.6)	48 (26.2)	182 (26.7)	20.9
7–8	166 (19.2)	53 (29.0)	113 (16.6)	31.9
≥ 9	85 (9.8)	27 (14.8)	58 (8.5)	31.8
Marital status:				
Single	209 (24.1)	39 (21.3)	170 (25.0)	18.7
Married	549 (63.5)	120 (65.6)	429 (62.9)	21.9
Divorced/Widowed/Separated	107 (12.4)	24 (13.1)	83 (12.1)	22.4
Occupation:				
Farmer	417 (48.2)	77 (42.1)	340 (49.9)	18.5
Housewife	97 (14.2)	25 (13.7)	72 (10.6)	25.8
Employee	94 (13.7)	28 (15.3)	66 (9.7)	29.8
Merchant	119 (17.4)	32 (17.5)	87 (12.8)	26.9
Student	95 (13.9)	16 (8.7)	79 (11.6)	16.8
Laborer	43 (6.3)	5 (2.7)	38 (5.6)	11.6
Educational status:				
Unable to read and write	384 (44.4)	60 (32.8)	324 (47.5)	15.6
Primary education	220 (25.4)	57 (31.1)	163 (23.9)	25.9
Secondary education	162 (18.7)	39 (21.3)	123 (18.0)	24.1
College and above	99 (11.5)	27 (14.8)	72 (10.6)	27.3
Graduated family member:				
Present	277 (32.0)	84 (45.9)	193 (28.3)	30.3
Absent	588 (68.0)	99 (54.1)	489 (71.7)	16.8
Home distance in walking hr:				
Up to 1 h	642 (74.2)	127 (69.4)	515 (75.5)	19.8
> 1 h	223 (25.8)	56 (30.6)	167 (24.5)	25.1
Counseling during purchase:				
Yes	375 (43.4)	44 (24)	331 (48.5)	11.7
No	490 (56.6)	139 (76)	351 (51.5)	28.4
Summary of knowledge level of respondents:				
Very high and High (≥ 80%)	151 (17.4)	11 (6.0)	140 (20.5)	7.3
Average (60–79%)	282 (32.6)	47 (25.7)	235 (34.5)	16.7
Low (40–59%)	255 (29.5)	75 (41.0)	180 (26.4)	29.4
Very low (< 40%)	177 (20.5)	50 (27.3)	127 (18.6)	28.2
Medications stored at home doesn’t need any special attention compared to other household materials.				
Strongly agree	39 (4.5)	10 (5.5)	29 (4.3)	25.6
Agree	107 (12.4)	33 (18.0)	74 (10.8)	30.8
I do not know	57 (6.6)	12 (6.6)	45 (6.6)	21.0
Disagree	432 (49.9)	87 (47.5)	345 (50.6)	20.1
Strongly disagree	230 (26.6)	41 (22.4)	189 (27.7)	17.8
Stored antimicrobials need a special attention compared to other medications.				
Strongly agree	162 (18.7)	32 (17.5)	130 (19.1)	19.8
Agree	284 (32.8)	46 (25.1)	238 (34.9)	16.2
I do not know	171 (19.8)	46 (25.1)	125 (18.3)	26.9
Disagree	200 (23.1)	52 (28.4)	148 (21.7)	26.0
Strongly disagree	48 (5.5)	7 (3.8)	41 (6.0)	14.6
It is being wise to keep antimicrobials at home as a reserve.				
Strongly agree	88 (10.2)	23 (12.6)	65 (9.5)	26.1
Agree	245 (28.3)	83 (45.3)	162 (23.8)	33.9
I do not know	40 (4.6)	9 (4.9)	31 (4.5)	22.5
Disagree	357 (41.3)	49 (26.8)	308 (45.2)	13.7
Strongly disagree	135 (15.6)	19 (10.4)	116 (17.0)	14.1
When family member get sick, it is better to use antimicrobials stored at home before going to health care institutions.				
Strongly agree	64 (7.4)	15 (8.2)	49 (7.2)	23.4
Agree	185 (21.4)	54 (29.5)	131 (19.2)	29.2
I do not know	74 (8.6)	29 (15.8)	45 (6.6)	39.2
Disagree	387 (44.7)	64 (35.0)	323 (47.4)	16.5
Strongly disagree	155 (17.9)	21 (11.5)	134 (19.6)	13.5
Antimicrobial resistance has nothing to do with storage of antimicrobials elsewhere at home or in the environment.				
Strongly agree	86 (9.9)	9 (4.9)	77 (11.3)	10.5
Agree	120 (13.9)	23 (12.6)	97 (14.2)	19.2
I do not know	353 (40.8)	92 (50.3)	261 (38.3)	26.1
Disagree	215 (24.9)	45 (24.6)	170 (24.9)	20.9
Strongly disagree	91 (10.5)	14 (7.6)	77 (11.3)	15.4

^a^ numbers in brackets represent the respective percentages

### Predictors of storage of antimicrobials at home

Predictors of home stored antimicrobials were determined as shown in Table 3. Predictors of home storage of antimicrobials with corresponding p-value were: age (0.002), family size (0.001), education status (< 0.001), home distance from the nearby healthcare institution (0.004), counseling while obtaining antimicrobials (< 0.001), knowledge level on antimicrobials (< 0.001), and perception of home storage of antimicrobials as a wisdom (0.001).

**Table 3:** Predictors of storage of antimicrobials at home at Mecha demographic surveillance and field research center, Ethiopia, March to May 2018 (N = 865)

Variables and categories	Storage		Crude odds ratio (COR)		Adjusted odds ratio (AOR)	
	Yes	No	COR (95% CI)	p-value	AOR (95% CI)	p-value
Place of residence:						
Rural	84	351	1.0			
Urban	99	331	1.25 (0.90–1.73)	0.182		
Sex:						
Male	71	252	1.08 (0.77–1.51)	0.646		
Female	112	430	1.0			
Age:						**0.002**
< 25	29	182	1.0		1.0	
25–34	43	157	1.72 (1.02–2.88)	0.040	2.45 (1.31–4.58)	**0.005**
35–44	60	172	2.19 (1.34–3.57)	0.002	3.36 (1.81–6.23)	**< 0.001**
45–54	28	89	1.97 (1.10–3.52)	0.021	3.35 (1.66–6.76)	**0.001**
> 54	23	82	1.76 (0.96–3.22)	0.067	3.43 (1.61–7.33)	**0.001**
Family size:						**0.001**
1–2	14	121	1.0		1.0	
3–4	41	208	1.70 (0.89–3.25)	0.106	1.31 (0.63–2.72)	0.470
5–6	48	182	2.28 (1.20–4.31)	0.011	1.58 (0.76–3.27)	0.222
7–8	53	113	4.05 (2.13–7.70)	< 0.001	3.22 (1.54–6.72)	**0.002**
≥ 9	27	58	4.02 (1.96–8.24)	< 0.001	2.97 (1.29–6.83)	**0.01**
Marital status						
Single	39	170	1.0			
Married	120	429	1.22 (0.81–1.82)	0.334		
Divorced/Widowed/Separated	24	83	1.26 (0.71–2.23)	0.428		
Occupation:						
Farmer	77	340	1.72 (0.66–4.51)	0.270		
Housewife	25	72	2.63 (0.93–7.44)	0.067		
Employee	28	66	3.22 (1.94–9.04)	0.026		
Merchant	32	87	2.79 (1.01–7.72)	0.048		
Student	16	79	1.53 (0.52–4.51)	0.432		
Laborer	5	38	1.0			
Educational status:						**< 0.001**
Unable to read and write	60	324	1.0		1.0	
Primary education	57	163	1.89 (1.25–2.84)	0.002	3.98 (2.44–6.51)	**< 0.001**
Secondary education	39	123	1.71 (1.09–2.70)	0.020	9.62 (4.94–18.74)	**< 0.001**
College and above	27	72	2.02 (1.20–3.41)	0.008	8.19 (4.09–16.40)	**< 0.001**
Graduated family member:						
Present	84	193	2.15 (1.53–3.00)	<0.001		
Absent	99	489	1.0			
Home distance in walking hr:						
Up to 1 h	127	515	1.0		1.0	
> 1 h	56	167	1.36 (0.95–1.95)	0.094	1.99 (1.25–3.18)	**0.004**
Counseling during purchase:						
Yes	44	331	1.0		1.0	
No	139	351	2.98 (2.0–4.31)	< 0.001	2.41 (1.54–3.89)	**< 0.001**
Knowledge on antimicrobials:						**< 0.001**
Very high and High (≥ 80%)	11	140	1.0		1.0	
Average (60–79%)	47	235	2.54 (1.28–5.07)	0.008	2.67 (1.26–5.61)	**0.01**
Low (40–59%)	75	180	5.30 (2.71–10.36)	< 0.001	2.85 (2.26–10.38)	**< 0.001**
Very low (< 40%)	50	127	5.01 (2.50–10.04)	< 0.001	3.82 (1.71–8.54)	**0.001**
Medications are stored like any household material.						
Strongly agree	10	29	1.59 (0.72–3.51)	0.250		
Agree	33	74	2.05 (1.21–3.49)	0.008		
I do not know	12	45	1.23 (0.59–2.52)	0.575		
Disagree	87	345	1.16 (0.77–1.75)	0.473		
Strongly disagree	41	189	1.0			
Stored antimicrobials need a special attention compared to other medications.						
Strongly agree	32	130	1.44 (0.59–3.51)	0.420		
Agree	46	238	1.13 (0.47–2.67)	0.778		
I do not know	46	125	2.15 (0.90–5.14)	0.084		
Disagree	52	148	2.06 (0.86–4.87)	0.101		
Strongly disagree	7	41	1.0			
It is being wise to keep antimicrobials at home as a reserve.						**0.001**
Strongly agree	23	65	2.16 (1.09–40.26)	0.026	1.83 (0.87–3.87)	0.113
Agree	83	162	3.13 (1.80–5.43)	< 0.001	2.62 (1.41–4.89)	**0.002**
I do not know	9	31	1.77 (0.73–4.30)	0.206	1.32 (0.49–3.65)	0.588
Disagree	49	308	0.97 (0.55–1.72)	0.920	1.05 (0.56–1.95)	0.889
Strongly disagree	19	116	1.0			
When family member get sick, it is better to use stored antimicrobials before going to healthcare institutions.						
Strongly agree	15	49	1.95 (0.93–4.09)	0.076		
Agree	54	131	2.63 (1.50–4.59)	0.001		
I do not know	29	45	4.11 (2.13–7.92)	< 0.001		
Disagree	64	323	1.26 (0.74–2.15)	0.388		
Strongly disagree	21	134	1.0			
Antimicrobial resistance has nothing to do with storage of antimicrobials elsewhere at home or in the environment.						
Strongly agree	9	77	0.64 (0.26–1.57)	0.333		
Agree	23	97	1.30 (0.63–2.70)	0.475		
I do not know	92	261	1.94 (1.04–3.59)	0.035		
Disagree	45	170	1.45 (0.75–2.81)	0.263		
Strongly disagree	14	77	1.0			

Age groups 25–34, 35–44, 45–54, and ≥ 55 were 2.45 [AOR: 2.45; 95%CI (1.31–4.58)], 3.36 [AOR: 3.36; 95%CI (1.81–6.23)], 3.35 [AOR: 3.35; 95%CI (1.66–6.76)], 3.43 [AOR: 3.43; 95%CI (1.61–7.33)] times more likely to store antimicrobials at home than the age group < 25 years, consecutively. Those who had a family size 7–8 and ≥ 9 were 3.22 [AOR: 3.22; 95%CI (1.54–6.72)] and 2.97 [AOR: 2.97; 95%CI (1.29–6.83)] times more likely to store antimicrobials at home than those with a family size of 1–2. Educational status of primary, secondary, and tertiary levels were: 3.22 [AOR:3.22; 95%CI (2.43–6.61)], 9.62 [AOR: 9.62; 95%CI (4.94–18.74)], and 8.19 [AOR: 8.19; 95%CI (4.09–16.40)] times more likely to store antimicrobials at home than those who couldn’t read and write, consecutively. Those respondents with a walking distance of > 1 h from the nearest health facility were 1.99 [AOR: 1.99; 95%CI (1.25–3.18)] times more likely to store antimicrobials at home than those who were in < 1 h walking distance. Respondents who had not received counseling during obtaining antimicrobials were 2.41 [AOR: 2.41; 95%CI (1.54–3.78)] times more likely to keep antimicrobials at home than those who received counseling. In this study knowledge on antimicrobials was assessed based on the summary of respondents’ responses given to 10 questions focused only on antimicrobials rather than considering responses on individual questions as shown in Table 4. Respondents with knowledge level average, low, and very low were 2.67 [AOR:2.67; 95%CI (1.26–5.61)], 2.85 [AOR: 2.85; 95%CI (2.26–10.38)], and 3.82 [AOR: 3.82; 95%CI (1.71–8.54)] times more likely to store antimicrobials at home compared to respondents category comprising very high and high knowledge level, consecutively. Participants who agreed with a statement that says storage of antimicrobials at home as a reserve is being wise, were 2.62 [AOR: 2.62; 95%CI (1.41–4.89)] times more likely to store antimicrobials compared to those who strongly disagree on the statement.

**Table 4:** Summary of question items and frequency of correct and incorrect responses of respondents which used for Likert scale categorization of knowledge level of respondents

Items	FoCRR	FoIRR
What are antimicrobial agents/drugs?	415	443
Medications can be kept at home like any other household materials?	573	292
Disposal of medications kept at home is similar to disposal of any household trash?	402	463
Any available medication at home is used to treat infectious diseases?	480	385
Antimicrobials medications are drugs used to treat all types of disease ?	411	454
Antimicrobials are effective drugs in common cold?	387	478
There is no special worry on keeping antimicrobial medications at home compared to any other medications?	361	504
Keeping antimicrobial medications elsewhere in the house or environment is a risk for development of microbial resistance?	697	168
Presence of unused antimicrobial medications elsewhere in the house or environment may affect human health?	529	336
Keeping antimicrobial medication(s) at home elsewhere can contaminate soil and water bodies?	402	463

FoCRR: frequency of correct response respondents; FoICRR: frequency of incorrect response respondents

## Discussion

The aim of this study was to assess home storage of antimicrobials and identify factors associated with storage practice. The information obtained from this study is of importance to healthcare professionals, regulatory bodies, and the community to draw strategies that help to reduce risks associated with home storage of antimicrobial and their inappropriate use. Evidences showed that most home stored antimicrobials are results of discontinuation of prescribed or self-initiated therapy.

### Stored antimicrobials at home

In the present study, more than one-fifth (21.2%) of surveyed households had home stored antimicrobials. This value was comparable with a study done in Tigray region of Ethiopia [19] and Iraq [6], though the former report included medications on current use as stored. However, it was lower than values reported from Uganda, Poland, China, and Spain [3, 20–22]. These variations could be due to the difference in the study population and variations in implementation of drug utilization guidelines and controlling strategies these countries follow. Commonly reported factors contributing for development of antimicrobial resistance were: patients’ poor adherence to prescribed antibiotics, widespread or overuse of antibiotics and broad-spectrum antibiotics use [23, 24].

Although, the primary source of most antimicrobials was prescription (69.2%), discontinuation of therapy (70.7%) comprised from symptomatic improvement (48.1%) and missing doses (22.6%) was the most common immediate source of home stored antimicrobials. Previous reports confirmed that the source of majority of antimicrobials are prescribed by health professionals for treatment of diagnosed illnesses to be used at home [17, 25–27]. Comparable discontinuation rate of 67.9% was reported in Gondar, Ethiopia [28]. However, a study in Yemen, Saudi Arabia, and Uzbekistan had reported a lower rate (49.0%) of discontinuation [29]. The higher level of discontinuation and retaining could be associated with poor knowledge on appropriate utilization and disposal, and lack of adequate counselling by health professionals. This condition indicates existence of vast adherence problems and high probability of irrational use of antimicrobials in the community that could be a risk for development of antimicrobial resistance due to insufficient doses and short duration of therapy. Studies showed that storage of unused antimicrobials at home with poor knowledge of disposal and storage could contaminate the environment [30] and initiate self-medication practice that has been reported as a risk factor for development of antimicrobial resistance [31–33].

As a group antimicrobials accounted two-third (66.5%) of all medications kept, which was comparable with a value reported at Basrah, Iraq [34]. The majority of stored antimicrobials were; amoxicillin (30.3%), cotrimoxazole (13.5%), metronidazole (12.0%), and ampicillin (9.6%) which was parallel with stored antimicrobials reported elsewhere [17]. This similarity could be related to the use of these antimicrobials commonly in the studied populations. Capsules followed by tablets were the most common stored preparations. This is similar to the findings from Tigray [19], Uganda [3], and Philippines [35]. Increased storage of these antimicrobials is related to their formulations since they are convenient for prolonged storage and easy for self-administration. This study showed that the most common site (90.2%) for placement of antimicrobials were shelves, tables, boxes and hand bags. Similar sites of placement had been reported in Tigray region of Ethiopia [19] and Qatar [5]. These sites are places to put many other household materials allowing access of people possibly every day leading to loss of their efficacy and safety, high potential of environmental exposure and risk of ingestion by children.

The present study showed that more than one-third (37.7%) of the households stored antimicrobials at home with objective of future use that could indicate extent of self-medication among households found to retain antimicrobials. This value was higher than reported to all types of medications in Tigray region of Ethiopia (10%), Qatar (4.0%), Uganda (21.6%) and Iraq (23.0%) [3, 7, 17, 19]. These varaitions could arise from differences of studied drug types and/or study population differences. More than one-third (35.1%) of antimicrobials were either expired or their expiry date is invisible. This finding is much higher than reported in Iraq and Tigray region of Ethiopia [17, 19]. This difference could be associated with differences in the category of medications assessed in these studies since the present finding was based only on antimicrobials compared to all type of mediations studied in Tigray and Iraq and/or due to long period storage practice of antimicrobials for future use in the community where this study was conducted.

Predictors of storage of antimicrobials at home identified this study were: age, family size, education status, home distance from the nearby healthcare institution, counseling during obtaining antimicrobials, knowledge level on antimicrobials, and perception of storage of antimicrobials as a wisdom.

Age groups 25–34, 35–44, 45–54, and ≥ 55 store antimicrobials at home more compared to age groups < 25 years old. A similar finding has been reported in China [36, 37]. In addition to storage, age was reported as one of the predictors of self-medication in a study elsewhere [38]. As age increases, experience to medical practices increases to the extent of practicing self-medication for which storage could be needed. Households with larger family size (≥ 7) were more likely to store antimicrobials at home compared to households with small family size (1–2). This could be associated with higher chance of getting sick as family members increases which could in turn increase retention or higher chance of sharing drugs in the family as suggested elsewhere [3, 39, 40]. When home distance to the nearest healthcare institution took >1hr by walking on feet, storage of antimicrobials at home was more likely than those households requiring up to 1hr walking time. This could be associated with users’ desire to minimize their effort by keeping drugs for family use as reported elsewhere [3, 41]. Respondents who attended primary, secondary, and tertiary educational were more likely to keep antimicrobials at home compared to those unable to read and write. In support of our finding, higher rate of keeping practice of a stock of antibiotics at home had been reported when the family has educated family member or a family member working in health related field [3, 37, 42, 43]. Although education enhances acquisition of information on antimicrobials, the information could initiate users to practice self-medication without having a clear and sufficient understanding of utilization of antimicrobials. Thus, to realize family healthcare interest educated people could keep antimicrobials obtained through different outlets.

Lack of/poor counseling about antimicrobial during obtaining them could lead users to discontinue medications and to keep them for future use for infections to which they did not have sufficient knowledge such as common cold. These suggestions could support our finding that respondents who reported nonattendance of counseling on medications during purchase were more likely to keep the drugs at home than those who received counselling as suggested elsewhere [44, 45]. Around 50% the respondents’ antimicrobial knowledge level was below average score which was comparable to those reported elsewhere [46, 47]. Respondents with antimicrobial knowledge level of average, low, and very low were identified to store antimicrobials more likely compared to respondents with high and very high antimicrobial level of knowledge. This finding could seemingly appear somewhat confusing when looked together with above finding which states, respondents who completed primary school and above stored antimicrobials more than respondents unable to read and write. However, as households comprise a family member who knows more and more about antimicrobials the tendency to store could be reduced as reflected elsewhere [48]. Thus, specific antimicrobial knowledge should not be confused with general awareness level. A review study had also reported that knowledge had mixed effects on antibiotic use behaviors [49]. In Ethiopia, the use of prescription-only medications including antimicrobial agents without medical consult has become alarmingly high [50]. Self-medication practice has been reported to be associated with educational status and level of knowledge on antimicrobials [10]. In addition, It has been concluded that self-medication has complex drivers, comprising of socio-economic factors, insufficient access to health care and poor implementation of regulatory policies on antimicrobials [51]. Respondents who perceived that reserving antimicrobials at home is being wise were more likely to keep the drugs compared to those who didn’t accept reserving as a wisdom. Similar findings have reported on the effect of perception in using stored medications at home without time limit [40, 52]. This could possibly related to their believe to safeguard family members’ health.

#### Strength of the study

besides using structured questionnaire, data on stored antimicrobials at home was collected by direct observation from house-to-house, which is expected to increase quality of the data and the study.

#### Limitation of the study

assessment of respondents perceptions through quantitative study may not show the actual scenario related with practice of home storage of antimicrobials. Besides, there could be recall bias of respondents on some questions that require recalling.

## Conclusion

Substantial proportion of households stored antimicrobials at home with storage conditions that favor or expose microbes in the surrounding environment to selection pressure. The immediate source of stored antimicrobials at home is discontinuation of prescribed or self-initiated therapy. Predictors of home storage of antimicrobials are age, family size, educational status, level of knowledge on antimicrobials, perception of home storage as a wisdom, counseling service during dispensing and distance of healthcare facilities. Strategies aiming to reduce home storage of antimicrobials in the community should give attention to age, family size, educational status, level of knowledge on antimicrobials, value given to storage, counseling services and distance from healthcare facilities. Furthermore, information should be provided on safe storage and disposal of antimicrobials at home.

## Data Availability

The structured questionnaire, datasets, and analysis output this study are available on request to the corresponding author.
